# Suppression of behavioral activity and hippocampal noradrenaline caused by surgical stress in type 2 diabetes model mice

**DOI:** 10.1186/s12868-020-0556-y

**Published:** 2020-02-17

**Authors:** Momoka Nishimura, Yuki Nomura, Moritoki Egi, Norihiko Obata, Makoto Tsunoda, Satoshi Mizobuchi

**Affiliations:** 1 Division of Anesthesiology, Department of Surgery Related, Kobe University Graduate School of Medicine, 7-5-2, Kusunoki-cho, Chuo-ku, Kobe, Hyogo 650-0017 Japan; 2 Graduate School of Pharmaceutical Sciences, University of Tokyo, 7-3-1, Hongou, Bunkyo-ku, Tokyo 113-0033 Japan

**Keywords:** Surgical stress, Type 2 diabetes mellitus, Hyperactive behavior, Postoperative hypoactivity

## Abstract

**Background:**

There has been much discussion recently about the occurrence of neuropsychological complications during the perioperative period. Diabetes is known to be one of the metabolic risk factors. Although the number of patients with diabetes mellitus (DM) has been increasing, the pathophysiology of postoperative neuropsychological dysfunction in DM patients is still unclear. Recently, a deficiency of neurotransmitters, such as monoamines, was reported to be associated with mental disorders. Therefore, we investigated the effects of surgical stress on behavioral activity and hippocampal noradrenaline (NA) level in type 2 diabetes mellitus model (T2DM) mice.

**Methods:**

Eighty-four 6-week-old male C57BL/6J mice were divided into four groups (non-diabetes, non-diabetes with surgery, T2DM, and T2DM with surgery groups). T2DM mice were established by feeding a high-fat diet (HFD) for 8 weeks. At 14 weeks of age, fifteen mice in each group underwent a series of behavioral tests including an open field (OF) test, a novel object recognition (NOR) test and a light–dark (LD) test. In the surgery groups, open abdominal surgery with manipulation of the intestine was performed 24 h before the behavioral tests as a surgical stress. Hippocampal noradrenaline (NA) concentration was examined in six mice in each group by high-performance liquid chromatography. The data were analyzed by the Mann–Whitney U test, and p values less than 0.05 were considered significant.

**Results:**

The T2DM group showed significantly increased explorative activity in the NOR test (P = 0.0016) and significantly increased frequency of transition in the LD test (P = 0.043) compared with those in the non-diabetic group before surgery. In T2DM mice, surgical stress resulted in decreased total distance in the OF test, decreased explorative activity in the NOR test, and decreased frequency of transition in the LD test (OF: P = 0.015, NOR: P = 0.009, LD: P = 0.007) and decreased hippocampal NA (P = 0.015), but such differences were not observed in the non-diabetic mice.

**Conclusions:**

Mice with T2DM induced by feeding an HFD showed increased behavioral activities, and surgical stress in T2DM mice caused postoperative hypoactivity and reduction of the hippocampal NA level.

## Background

There has been much discussion recently about the occurrence of neuropsychological complications during the perioperative period [1]. Risk factors for neuropsychological complications including obesity, diabetes, and dyslipidemia have been reported [2]. The number of people with diabetes mellitus (DM) worldwide has increased to about 425 million [3] and over 90% of those people have type 2 diabetes mellitus (T2DM) caused by diet and lifestyle [4]. In the early postoperative period, cognitive outcome is worse in DM patients than in non-diabetic patients [5], and transient advanced mental impairment occurs more frequently in DM patients after aortic surgery [6]. T2DM is also well known as one of the risk factors for postoperative delirium [7–9]. However, the pathophysiology of neuropsychological disorders and postoperative behavioral alterations in DM patients has remained unclear. Since the number of DM patients undergoing surgery will continue to increase, it is important to investigate the neuropsychological effect of surgical stress in DM patients.

Recently, it has been reported that monoamine, one of the neurotransmitters, plays very important roles in arousal, cognitive activity and emotional activity [10]. Dysfunction of the monoamine system was shown to be associated with mental illnesses including depression [11], affective disorder [12] and anxiety disorder [13]. From a psychopathological point of view, we hypothesized that neurotransmitter deficiency is associated with postoperative behavioral alterations and mental impairment in DM patients.

In the monoamine system, noradrenaline (NA) has been shown to be associated with responses to stressful events and to be activated in an unexpected condition [10]. The locus caeruleus projection of NA to the cortex and hippocampus is thought to be strongly involved in modulation of attention [14]. In animal models, an increase of the hippocampal NA signal has been shown to be associated with neuronal plasticity [15], synaptic plasticity [16], and memory retrieval [17], while a decrease of hippocampal NA levels has been shown to be related to anxiety [18], depression [19] and hypoactivity [20]. However, the effect of acute surgical stress on the hippocampal NA system is not known.

Therefore, we focused on behavioral alterations and hippocampal NA during the perioperative period in the state of DM. We used adult mice in which T2DM was induced by feeding a high-fat diet (HFD) [21] in order to evaluate the effect of T2DM on perioperative behavioral alterations. The purpose of this study is to clarify the impact surgical stress has on postoperative behavioral activity and hippocampal NA in T2DM model mice.

## Results

### Mild chronic hyperglycemia in T2DM mice

**Fig. 1:**
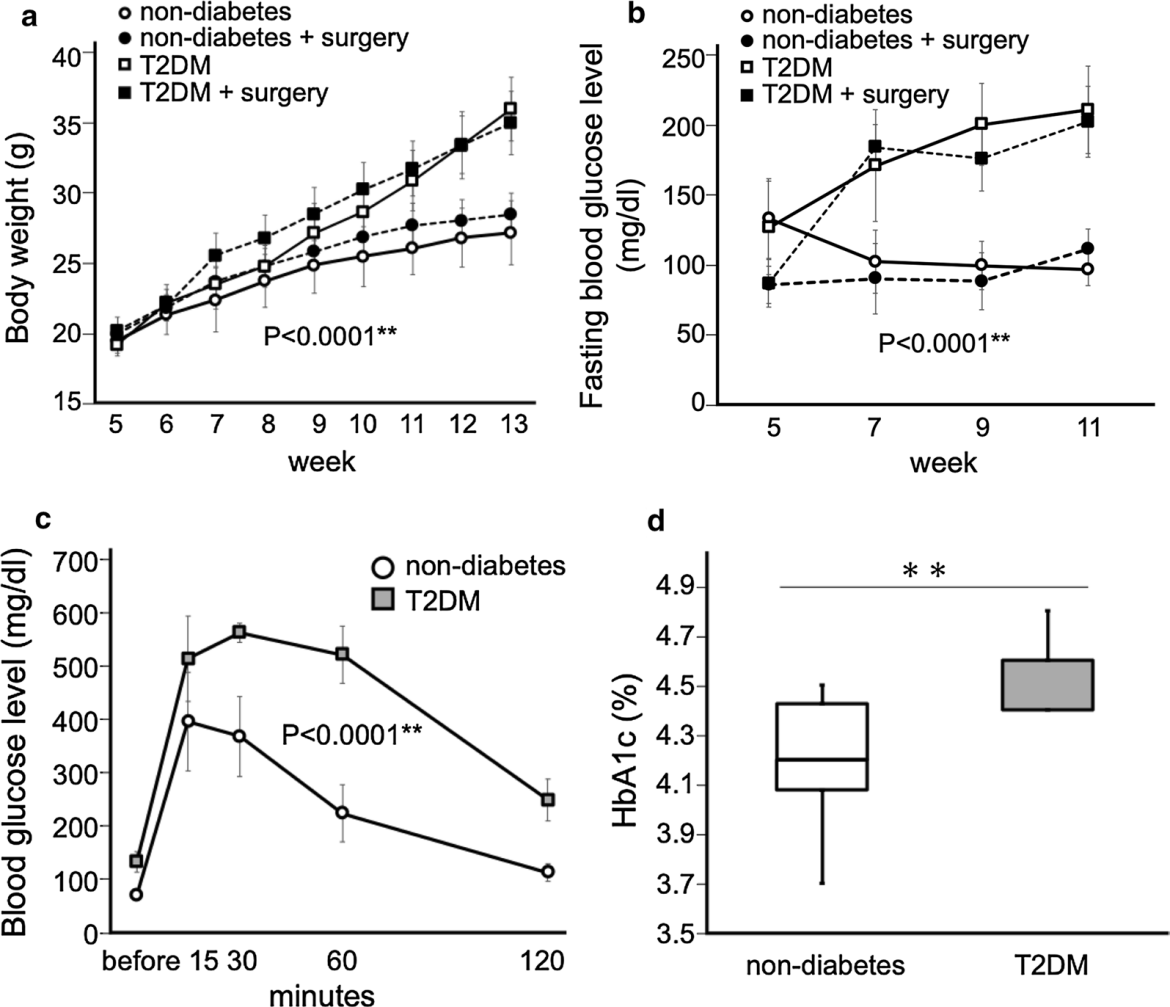
Biophysical parameters in the T2DM model mice. **a** Comparison of body weight gains in each group. The body weight gain of the T2DM group was significantly greater than that of the non-diabetic group. **b** The fasting blood glucose level in the T2DM group was significantly higher than that in the non-diabetic group. **a**, **b** The line with white circles indicates the non-diabetes and the wave line with black circles indicated the non-diabetes with surgery. The line with white squares indicates the T2DM and the wave line with black squares shows the T2DM with surgery. The results are shown as mean ± standard deviation, n = 12 per group. **c** In the IPGTTs, the glucose level in the T2DM group was significantly higher than that in the non-diabetic group, and prolonged hyperglycemia was observed in the T2DM group. The line with white circle showed the non-diabetic group and the line with gray square indicated the T2DM group. The results are shown as mean ± standard deviation, n = 12 per group. **d** The HbA1c level in the T2DM group was higher than that in the non-diabetic group. The white bar indicates the non-diabetic group and the gray bar indicates the T2DM group. The results are shown as medians ± quartile, n = 10 per group. **P < 0.01

Figure 1 shows that the T2DM model was established by feeding an HFD for 8 weeks (Fig. 1a–d). A graph of body weight gain is shown in Fig. 1a. Body weight in the T2DM group was significantly increased compared with that in the non-diabetic group (n = 12 per group, P < 0.0001). Changes in the levels of fasting blood glucose are shown in Fig. 1b. Fasting blood glucose level in the T2DM group was significantly higher than that in the non-diabetic group (n = 12 per group, P < 0.0001). There was no difference on the transition graph of body weight and fasting blood glucose between the non-surgery and surgery group in both non-diabetic and T2DM model (Fig. 1a, b).

The results of intraperitoneal glucose tolerance tests (IPGTTs) performed at 13 weeks of age are shown in Fig. 1c. The glucose level in the T2DM group was significantly higher than that in the non-diabetic group and prolonged hyperglycemia was observed in the T2DM group (n = 12 per group, P < 0.0001), indicating that the glucose intolerance was caused by feeding an HFD. The level of hemoglobin A1c (HbA1c) in the T2DM group was also significantly higher than that in the non-diabetic group (n = 10 per group, P = 0.009) as shown in Fig. 1d. This result indicated that mild chronic hyperglycemia was sustained in T2DM model mice.

### Hyperactive behaviors of T2DM mice

**Fig. 2:**
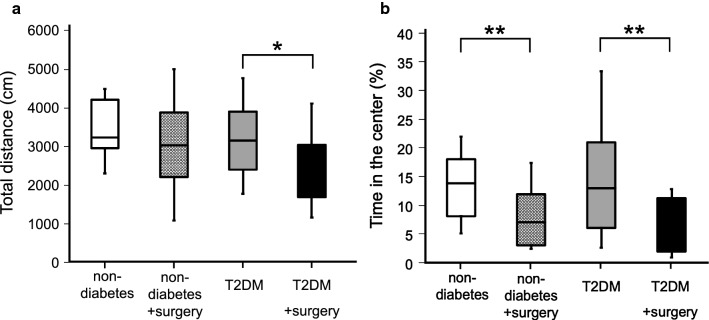
Results of the open field (OF) test. **a** Total distance in the OF test. The total distance was significantly shorter in the T2DM with surgery group than in the T2DM group. **b** Time spent in the center area in the OF test. The time spent in the center was significantly decreased after surgery in both the non-diabetic group and T2DM group. Each graph is shown as medians ± quartile, n = 15 per group. *P < 0.05, **P < 0.01

**Fig. 3:**
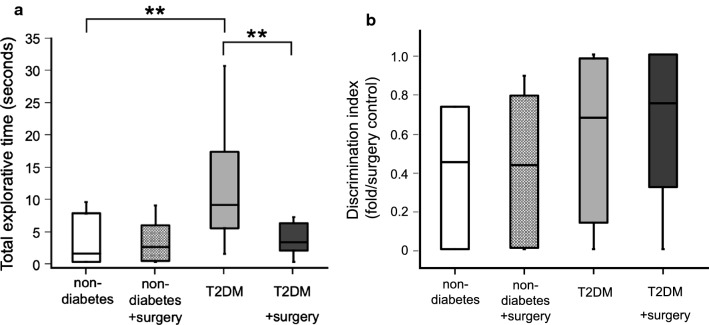
Results of the novel object recognition (NOR) test. **a** Total explorative time in the NOR test during the familiarization phase. The total explorative time was significantly longer in the T2DM group than in the non-diabetic group. However, the explorative activity was significantly decreased after surgery in T2DM mice. **b** Discrimination index in the NOR test. There was no difference between the non-diabetic group and T2DM group during the perioperative period. Each graph is shown as medians ± quartile, n = 15 per group. **P < 0.01

**Fig. 4:**
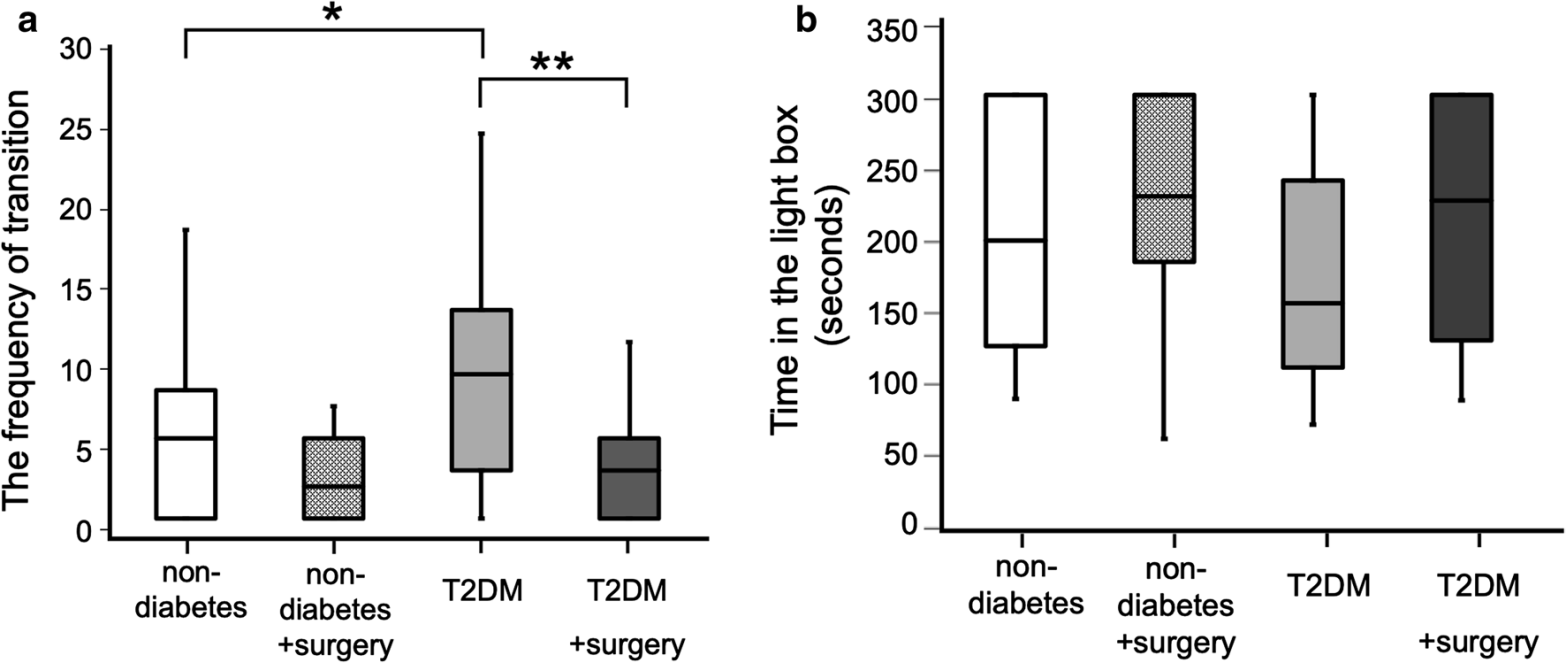
Results of the light–dark (LD) test. **a** Frequency of transition in the LD test. The number of transitions in the T2DM group was significantly larger than that in the non-diabetic group. However, the number of transitions significantly decreased after surgery in the T2DM mice. **b** Time spent in the light box. There was no difference between the non-diabetic group and the T2DM group during the perioperative period. Each graph is shown as medians ± quartile, n = 15 per group. *P < 0.05, **P < 0.01

A series of behavioral tests was performed at 14 weeks of age. The results of open field (OF) tests for evaluation of spontaneous locomotor activity and anxiety-like behaviors are shown in Fig. 2a, b. There were no differences in the total distance and time spent in the center in the OF test between the non-diabetic group and the T2DM group. Novel object recognition (NOR) tests were performed to evaluate explorative activity and cognitive function. Total explorative times during the familiarization phase of the NOR tests are shown in Fig. 3a. The time spent for explorative activity was significantly longer in the T2DM group than in the non-diabetic group (P = 0.001). In the testing phase, there was no difference in the discrimination index between the T2DM group and the non-diabetic group as shown in Fig. 3b, and cognitive dysfunction was therefore not revealed in this experiment. Light–Dark (LD) tests were performed to examine explorative activity and anxiety. The frequency of transition between the light and dark chambers was significantly higher in the T2DM group than in the non-diabetic group (P = 0.043) as shown in Fig. 4a. However, the time spent in the light chamber was not different between the non-diabetic group and the T2DM group as shown in Fig. 4b.

### Decrease of postoperative activity in T2DM mice caused by surgical stress

To evaluate postoperative behavioral alterations, the same series of behavioral tests was performed 24 h after open abdominal surgery in both non-diabetic with surgery group and T2DM with surgery group. The total distance significantly decreased after surgery in T2DM mice (Fig. 2a, P = 0.015) but not in the non-diabetic mice. The time spent in the center significantly decreased in both non-diabetic mice and T2DM mice after surgery (Fig. 2b, non-diabetes vs non-diabetes with surgery: P = 0.006, T2DM vs T2DM with surgery: P = 0.006), indicating that anxiety-like behavior was caused by surgical stress in both non-diabetic mice and T2DM mice. In the familiarization phase of the NOR test, the total explorative time markedly decreased after surgery in T2DM mice (Fig. 3a, P = 0.009) but not in non-diabetic mice (P = 0.802). In the testing phase, there was no difference in the discrimination index between non-diabetic mice and T2DM mice during the perioperative period (Fig. 3b), and cognitive dysfunction was therefore not revealed in either non-diabetic mice or T2DM mice after surgery. In the LD test, the frequency of transition between the light and dark chambers was significantly reduced after surgery in T2DM mice (Fig. 4a, P = 0.007). Surgical stress did not affect the time spent in the light chamber for either non-diabetic mice or T2DM mice (Fig. 4b).

### Decrease of hippocampal noradrenaline (NA) induced by surgical stress in T2DM mice

**Fig. 5:**
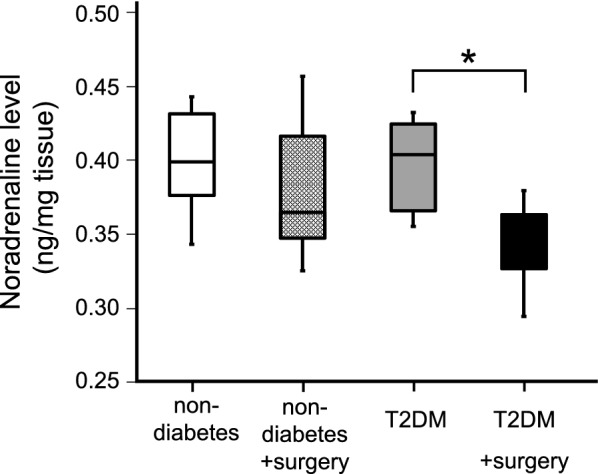
Noradrenaline concentration in the hippocampus determined by high-performance liquid chromatography. The hippocampal noradrenaline concentration was significantly decreased after surgery in T2DM mice. Each bar is shown as median ± quartile, n = 6 per group. *P < 0.05

High-performance liquid chromatography (HPLC) analysis was performed to examine the hippocampal NA level. There was no difference between the hippocampal NA levels in the non-diabetic and T2DM group before surgery (Fig. 5, P = 0.937). However, the hippocampal NA level was significantly reduced after surgery in T2DM mice (P = 0.015), while there was no difference in non-diabetic mice (non-diabetes vs non-diabetes with surgery, P = 0.485).

## Discussion

The purpose of the present study was to examine the effects of surgical stress on postoperative behavioral activity and hippocampal NA in T2DM model mice. In our study, T2DM model mice fed an HFD for 8 weeks showed an increase of explorative activity in the NOR test and more frequent transition in the LD test. In the postoperative period, no significant difference was observed in behavioral tests and the hippocampal NA between non-diabetes with surgery and T2DM with surgery. Focusing on T2DM, the activity was decreased after surgery compared to before surgery, and the hippocampal NA level also decreased after surgery. These finding may indicate that surgical stress had a suppressive effect on behavioral activity and the hippocampal NA in T2DM.

In our experiment, we used 14-week-old adult male mice after feeding an HFD for 8 weeks and we performed NOR, LD and OF tests for perioperative behavioral assessment. Before the operation, we found hyperactive behavioral alterations in T2DM mice in the NOR and LD tests. The NOR test was conducted to evaluate explorative activity for novel objects and cognitive function. Interestingly, our T2DM model mice showed an increase in total explorative time without cognitive dysfunction. Sims-Robinson et al. reported that 6-week-old male mice after feeding an HFD for 2 weeks showed increased explorative activity in the NOR test, being in agreement with our results [22]. In the LD test for assessing spontaneous explorative activity and anxiety, our T2DM mice showed an increase in the frequency of transition without a change in the time spent in the light chamber. Kurhe et al. reported that Swiss albino mice fed an HFD for 14 weeks, a duration that is 1.75-times longer than that for our T2DM mice, showed less frequent transition [23], and their results are different from our results. We considered that the length of the feeding period caused the difference in behavioral alterations. Furthermore, the T2DM mice in our study did not show anxiety in the LD test. It has been reported that anxiolytics increased the number of transitions between the light and dark compartments [24] and that an increase in transitions reflects anxiolytic activity [25]. We thought that our model mice showed hyperactive behavioral alterations because they did not have anxiety.

The open abdominal surgery used as surgical stress caused postoperative hypoactivity in the T2DM mice as indicated by the decrease in total distance in the OF test, decrease in explorative activity in the NOR test and decrease in the frequency of transition in the LD test. In the OF test, we evaluated locomotor activity and anxiety behavior. The surgical stress in T2DM mice caused a decrease in total distance moved compared with that before surgery, but there was no difference in control mice. Zhao et al. reported that 15-month-old male mice fed an HFD for 14 weeks did not show a decrease in total distance in the OF test after internal fixation surgery of a tibial fracture [26]. The discrepancy between our results and their results might have been caused by the differences in type of surgical stress, duration of HFD feeding and age of the mice. Regarding anxiety behavior, the time spent in the center decreased after surgery compared with that before surgery in both the T2DM and control groups. These results indicated that the surgical stress induced anxiety in mice. Our results are supported by the results of a study by Lu et al. showing that 16-month-old Sprague–Dawley rats one day after splenectomy exhibited anxiety-like behavior indicated by traveling less in the central area [27]. Moreover, in the present study, the T2DM mice after surgery showed a decrease of activity in all three behavioral tests. It has been reported that anxiety caused a decrease of transition frequency [28] and a decrease of explorative activity [29]. We considered that the anxiety state induced by surgical stress in the T2DM mice caused the postoperative hypoactivity in the behavioral tests.

Among neurotransmitters, it is well known that an increase of NA release in the brain is an acute response to stress [30] and that NA regulates cognition [31], motivation and social interactions [32]. In the hippocampus, a decrease of NA is associated with mental impairments such as a decrease of activity, depression and cognitive dysfunction [18–20, 33]. In the present study, hippocampal NA level was decreased 24 h after surgical stress. Regarding the effect of surgery on hippocampal NA, Xu et al. reported that rats with depression caused by olfactory bulbectomy showed a decrease of hippocampal NA two weeks after the surgery [34]. Liu et al. demonstrated that chronic unpredictable mild stress for 6 weeks caused depressive behaviors accompanied by a decrease of hippocampal NA in mice [35]. According to these findings, we considered that the surgical stress in the T2DM model mice induced the decrease of hippocampal NA and that it might be associated with suppression of behavior in the T2DM model mice after surgery. This might lead to elucidation of the pathophysiology of perioperative mental alterations in diabetic patients.

There are some limitations in the current study. First, we conducted the series of behavioral tests in the same mice in order to reduce the total number of mice, and we performed the tests within a limited postoperative period. However, some more behavioral experiments including assessments of cognitive function, anxiety and depression-like behavior should also be conducted to identify the postoperative behavioral changes in more detail. Second, we used open abdominal surgery as surgical stress and examined behaviors 24 h after the surgery in this study. However, our procedure may not exactly mimic postoperative surgical stress in patients with diabetes, since there are many kinds of surgical invasions and patients have some other complications in clinical situations. The Further experiments are needed to investigate the effects of different degrees of invasion and time-dependent effects on behavior and hippocampal NA in the T2DM mice. Finally, we did not observe downstream signaling of hippocampal NA, and it is therefore unclear how the change in hippocampal NA affected perioperative behavioral alterations in this study. Therefore, future research is required to prove it.

## Conclusions

In conclusion, we found that T2DM model mice fed an HFD for 8 weeks showed behavioral alterations and that surgical stress in the T2DM model mice caused postoperative hypoactivity and a decrease in hippocampal NA.

## Methods

### Animals

Four-week-old male C57BL/6J mice, weighting 15.7 (median) ± 2.7 g, were purchased from SLC Center Japan. We used only male mice to avoid the influence of sex hormones in the behavioral experiments. The animals were group-housed 3 per plastic cage at our animal experimental center until the behavioral tests in a temperature-(24 °C), humidity- and light-controlled room (12-h light–dark cycle) with ad libitum access to water and rodent chow according to the Kobe University standards for animal welfare.

All animal experiments and procedures were performed in accordance with the national institutional guidelines for proper conduct of animal experiments and complied with the international guiding principles for biomedical research involving animals. This study was approved by the Kobe University Animal Experiment Committee (approved on October 23, 2017, No. P151004).

### Experimental protocol

**Fig. 6:**
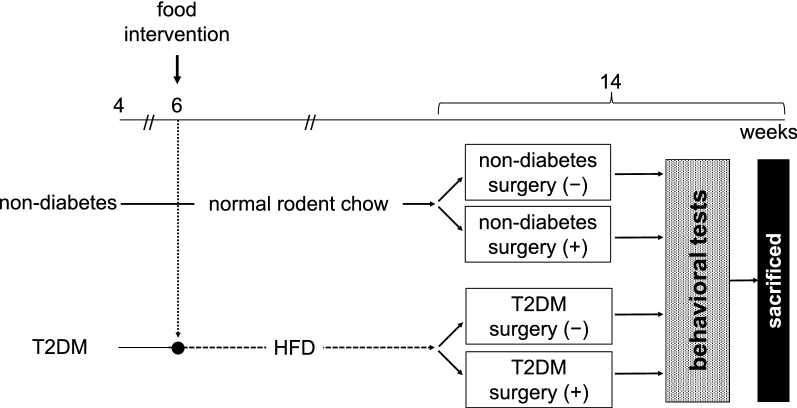
Experimental procedure in this study. *T2DM* type 2 diabetes mellitus, *HFD* high-fat diet

The aim of the current study was to determine the effect of surgical stress on T2DM model mice. Our experimental protocol is shown in Fig. 6. Eighty-four mice were randomly divided into four groups (n = 21 per group, non-diabetes, non-diabetes with surgery, T2DM, T2DM with surgery groups). Sample size was calculated with EZR [36] followed by the results of a preliminary behavioral experiment using the OF test for five mice in each of the T2DM and T2DM with surgery groups. In each group, fifteen mice were used for the behavioral tests, and six mice were used to examine the hippocampal NA level. The T2DM groups were fed HFD32 (32% fat content and 56.7% of fat kcal; CLEA Japan, Inc), and the non-diabetic groups were fed normal rodent chow. Diet intervention was started at 6 weeks of age and lasted for 8 weeks until 14 weeks of age. As biophysical parameters, we investigated body weight every week and fasting blood glucose level every 2 weeks. Glucose tolerance evaluation by IPGTTs was performed at 13 weeks of age, and HbA1c level was examined at 14 weeks of age. At 14 weeks of age, a series of behavioral tests was performed, and surgery was performed for the surgery groups 24 h before the behavioral tests. After the behavioral tests, mice were inhaled 6% sevoflurane and euthanized by opening chest and removing blood transcardially.

### Surgical procedure

Open abdominal surgery was performed in the surgical groups (non-diabetes with surgery and T2DM with surgery groups) according to a previously study [37]. The procedure was conducted at an animal raboatory (at 8:00 a.m.). General anesthesia was induced with 3.5% sevoflurane in 50% oxygen in a plastic chamber and was maintained with 3.5% sevoflurane (covering 1 minimal alveolar concentration (1 MAC) of adult C57BL/6J mice [38]) by using a mask during the surgical procedure. The concentrations of sevoflurane and oxygen in the inhalational anesthesia were continuously monitored by using anesthetic monitoring equipment (Datex, IMI Co., Ltd,). Each mouse was gently restrained to a heating pad (37 °C) during the procedure. After shaving the abdominal incision area, sterilization was provided by povidone iodine. A 1-cm vertical incision was made in the middle of the abdomen. During the surgery, the whole intestine was exteriorized from the peritoneal cavity, covered with moist gauze, and then manipulated with sterile cotton swabs for 1 min. Afterward, the peritoneum muscle and skin were repaired separately with 5-0 nylon (Natsume Seisakusho Co, Japan). Total anesthesia time was 20 min for each procedure. After the closing incision and 8 h later, EMLA^®^ cream (2.5% lidocaine and 2.5% prilocaine) was applied to treat the surgery-associated pain [39].

### Body weight and fasting blood glucose level

After food intervention, body weight was recorded every week and blood glucose level was examined every 2 weeks. The fasting glucose level was measured by Glutest Neo α (Sanwa Kagaku Kenkyusho, Japan) following 6 h of fasting [40].

### Intraperitoneal glucose tolerance tests (IPGTTs)

IPGTTs were performed at 13 weeks of age, 7 weeks after food intervention. The animals were fasted for 16 h prior to the glucose tolerance test. After measuring the basal level of blood glucose (0 min), each animal was injected with glucose solution (2 g glucose/kg body weight) intraperitoneally (i.p.) and blood glucose was measured at 15, 30, 60 and 120 min after the injection [40].

### Hemoglobin A1c (HbA1c)

At 14 weeks of age, blood samples were collected from the tail vein and HbA1c was measured by using a glycated hemoglobin A1c kit (DCA 2000^®^ HbA1c cartridge, Siemens Healthineers Japan).

### Behavioral tests

We performed a series of sequential behavioral tests to evaluate perioperative behaviors. For the surgical groups, behavioral tests were performed 24 h after the open abdominal surgery. Each mouse was isolated in an individual cage and transferred to the test room for habituation 1 h before the behavioral tests. The order of mice for the test was in a random manner, and the test chamber was cleaned after each session. Each behavioral test was automatically recorded. The behavioral tests were performed at 8:00 a.m. to 15:00 p.m. in the light phase. Details of the series of behavioral experiments are as follows.

### Open field test (OF test)

An open field test was used for measuring locomotor activity and anxiety [41]. The open field apparatus was constructed of a gray acrylic plate of 60 × 60 cm in size with wall of 40 cm in height. Each mouse was placed close to the wall of the apparatus and exposed to the novel environment, and activity was monitored for 5 min twice with a 1-h interval. Activity was automatically recorded by a video tracking system [42] and analyzed by using the SMART^®^ system (Bio Research Center Corporation, Japan). Repeated exposure to the open field apparatus has been shown to result in acclimation to the field for the NOR test [43].

### Novel object recognition test (NOR test)

The NOR test was performed to evaluate both explorative activity for objects and cognitive ability. One hour after the open field test, the NOR test was performed in the same apparatus. The test consisted of two phases: a familiarization phase and a testing phase. In the familiarization phase, two identical objects, glass beakers (5.5 cm in diameter × 7 cm in height), were placed 20 cm from each side wall of the chamber and 20 cm apart. The familiarization phase was performed for 5 min and each animal was returned to its home cage after the familiarization phase. In the testing phase 1 h after the familiarization phase, one of the familiar objects was replaced with a novel object, a plastic ball (7 cm in diameter). The testing phase was performed for 5 min. The time spent exploring each object was recorded and analyzed by the SMART^®^ system. Cognitive outcome was determined by the “discrimination index”. The discrimination index was calculated as (the time spent in the novel object zone)/(the time spent in familiar object zone + the time spent in novel object zone) [44].

### Light–dark test (LD test)

The LD test was performed 1 h after the NOR test to investigate spontaneous explorative activity and anxiety behavior. A new environment and light are known to be mild stressors, and an innate aversion response to the light chamber represents explorative and anxiety behavior of rodents [45]. The LD test apparatus consisted of bright and dark compartments (length of 18 cm × width of 30 cm × height of 16 cm) connected by a small opening (10 cm in diameter, semicircle shape). To evaluate the naive behavior of mice in the usual state without excessive stress, the light chamber was illuminated (200 lx), whereas the dark chamber was 1–2 lx. The LD test was performed for 5 min. The total number of transitions and the time spent in the light chamber were recorded on video.

### High-performance liquid chromatography (HPLC)

Hippocampal NA concentration was measured by HPLC-electrochemical detection. Hippocampal tissues of six mice in each group for evaluating NA were collected after beheading the mice and were homogenized in 0.4 M perchloric acid in a ratio of one part tissue to ten parts perchloric acid and centrifuged at 4000*g* for 20 min at 4 °C. Then the supernatant was collected and preserved at − 80 °C until sample analysis. Samples were purified by using MonoSpin^®^ (GL Sciences Inc. Japan). NA levels were measured by the HPLC system and 3,4-dihydroxybenzylamine was used as the internal standard. Respective peak and elution times of the samples were evaluated by relative comparison to the standard. Chromatographic separation was performed on an Inertsil ODS-4 column, 5 μm, 250 × 3.0 mm in I.D. (GL Sciences Inc, Japan). The column temperature was maintained at 35 °C. The flow rate was 0.5 mL/min. Injection volume was 20 μL. The mobile phase consisted of 20 mM acetate-citrate buffer and acetonitrile containing sodium 1-octanesulfonate.

### Statistical analysis

Statistical analysis by comparison between two groups (non-diabetes vs T2DM, non-diabetes with surgery vs T2DM with surgery, non-diabetes vs non-diabetes with surgery and T2DM vs T2DM with surgery) was conducted using EZR [36] and JMP Statistical Discovery™ (SAS Institute Inc, Japan). The data for the behavioral test and for the hippocampal NA and HbA1c levels were analyzed by the Mann–Whitney U test using EZR. Body weight, fasting blood glucose level and IPGTTs were subjected to two-way repeated measures analysis of variance (ANOVA) by using JMP. P values less than 0.05 were considered to indicate a significant difference. In the figures, the error bars of data are presented as medians ± quartile for the Mann–Whitney U test and mean ± SD for ANOVA.

## Data Availability

The raw datasets supporting the results of this study are available in the Mendeley Data, v1. 10.17632/dphnhn3kjw.1. http://dx.doi.org/10.17632/dphnhn3kjw.1. The datasets analyzed during the current study are available from the corresponding author on reasonable request.

## Abbreviations

ANOVA: Analysis of variance
HbA1c: Hemoglobin A1c
HFD: High-fat diet
HPLC: High-performance liquid chromatography
IPGTT: Intraperitoneal glucose tolerance test
NA: Noradrenaline
NOR: Novel object recognition
LD: Light–dark
OF: Open field
T2DM: Type 2 diabetes mellitus
